# Patient reported outcome instruments used in clinical trials of HIV-infected adults on NNRTI-based therapy: a 10-year review

**DOI:** 10.1186/1477-7525-11-164

**Published:** 2013-10-03

**Authors:** Kit N Simpson, Kristin A Hanson, Gale Harding, Seema Haider, Margaret Tawadrous, Alexandra Khachatryan, Chris L Pashos, Albert W Wu

**Affiliations:** 1 Medical University of South Carolina, 171 Ashley Avenue, Charleston, SC 29425, USA; 2 UBC: An Express Scripts Company, 185 Dorval Ave, Suite 500, Dorval, QC H9S 5J9, Canada; 3 Evidera, 7101 Wisconsin Avenue, Suite 600, Bethesda, MD 20814, USA; 4 Pfizer Inc., 558 Eastern Point Road, Groton, CT 06340, USA; 5 Pharmerit, 4350 East West Highway, Suite 430, Bethesda, MD 20814, USA; 6 UBC: An Express Scripts Company, 430 Bedford Street, Lexington, MA 02420, USA; 7 Johns Hopkins Bloomberg School of Public Health, 615 North Wolfe Street, Baltimore, MD 21205, USA

**Keywords:** HIV, Patient-reported outcome (PRO), Instrument, NNRTI

## Abstract

**Background:**

Patient-reported outcomes (PROs) may provide valuable information to clinicians and patients when choosing initial antiretroviral therapy.

**Objective:**

To identify and classify PRO instruments used to measure treatment effects in clinical trials evaluating NNRTIs.

**Methods:**

We conducted a structured literature review using PubMed to identify NNRTI trials published from March 2003 to February 2013. Studies identified--based on disease, instrument, PRO, and NNRTI medication terms were reviewed--to identify PRO instruments. Domains measured within each instrument were recorded to understand key areas of interest in NNRTIs.

**Results:**

Of 189 articles reviewed, 27 validated instruments were administered in 26 unique trials, with a mean of 1.9 instruments (median: 1; range: 1–7) per trial. The Medical Outcomes Study HIV Health Survey (MOS-HIV) was the most commonly used instrument (n = 8 trials). Seventeen trials (65%) included at least one multidimensional health-related quality of life (HRQL) instrument (HIV-targeted, n = 11; general, n = 8). Other validated instruments measured sleep (n = 5), depression (n = 5), anxiety (n = 4), psychiatric symptoms (n = 2), beliefs about HIV medications (n = 2), HIV symptoms (n = 1), and stress (n = 1).

**Conclusions:**

Although review of recent NNRTI trials suggests a lack of consensus on the optimal PRO instruments, a typical battery is comprised of a multidimensional HRQL measure coupled with one or more symptom measures. Further work is needed to clarify advantages and disadvantages of using specific PRO instruments to measure relevant constructs and to identify the most useful batteries of instruments for NNRTI trials.

## Background

The primary goal of HIV therapy is to increase disease-free survival and improve health-related quality of life (HRQL) by containing viral replication, avoiding drug resistance, and boosting immunologic function by restoring CD4 count [1,2]. The United States Department of Health and Human Services (DHHS) has recommended several preferred and alternative initial highly active antiretroviral therapy (HAART) regimens which have comparable efficacy, but different pharmacokinetic or pharmacodynamic properties. DHHS further recommends tailoring the HAART regimen to the patient--based on expected side effects, convenience, comorbidities, potential drug interactions, and results of any pre-treatment genotypic drug-resistance testing--to optimize medication adherence and improve long-term treatment success [3]. Since some of these constructs must be measured from the patient perspective, it is important to consider patient-reported outcomes (PROs) when selecting initial antiretroviral therapy.

A PRO is defined as any report of the status of a patient’s health condition that comes directly from the patient without interpretation of the patient’s response by a clinician or anyone else [4]. In clinical trials, PRO instruments can be used to measure the effect of a medical intervention on one or more concepts – such as symptoms, functioning, severity of disease, or HRQL. Given the armamentarium of potent HAART regimens available today, HIV infection has been transformed from a terminal illness into a chronic condition. As such, there is a strong case for evaluating the impact of antiretroviral therapies on broader aspects of patient’s lives, including psychological health and emotional adjustment. The majority of published comparative treatment studies that include PROs are limited to comparing differences between protease inhibitor (PI) and non-nucleoside reverse transcriptase inhibitor (NNRTI)-based regimens. This may be due in part to the fact that for several years, treatment guidelines have recommended initiating HAART with two NRTIs plus either an NNRTI or a boosted PI [5,6]. However, this broad comparison may miss important distinctions among regimens that are related to within-class PRO differences.

Although five NNRTIs have received Food and Drug Administration (FDA) approval to date, European AIDS Clinical Society (EACS) and DHHS treatment guidelines recommend efavirenz (EFV) as the NNRTI of choice to be used in most treatment-naïve HIV-infected adults initiating NNRTI-based therapy [3,6]. Other recommended NNRTIs include nevirapine (NVP) and rilpivirine (RVP). In the absence of head-to-head comparative clinical trials demonstrating clinical superiority of one NNRTI over another, PROs become an important tool for identifying treatment differences and informing treatment choices. A necessary first step to understanding differences among specific NNRTIs is to examine the PRO instruments being used in clinical trials and the aspects of health they measure. Therefore, the purpose of this study was to identify and classify PRO instruments used to measure treatment effects in clinical trials evaluating NNRTIs.

## Methods

### Literature search

An electronic search using PubMed was conducted evaluating studies published from March 2003 to February 2013. Our search strategy included a combination of Medical Subject Headings (MeSH) terms for HIV [HIV OR HIV infections], MeSH terms associated with PROs/instruments [questionnaires OR interviews as topic OR quality of life OR patient satisfaction OR self-evaluation programs], Substance Names of NNRTIs [efavirenz OR nevirapine OR delavirdine OR etravirine OR rilpivirine OR efavirenz, emtricitabine, tenofovir disoproxil fumarate drug combination], and clinical trial Publication Types [clinical trial OR clinical trial, phase IV OR clinical trial, phase III OR clinical trial, phase II OR controlled clinical trial OR randomized controlled trial]. A complete list of all search terms used, including terms used in title/abstract searches, is shown in Additional file 1. We limited our search to articles written in English with abstracts available. In addition to the PubMed search, we conducted a manual search of the bibliographies of the electronically-identified primary studies and review articles.

### Study selection

The inclusion and exclusion criteria for studies to be considered in our systematic review were established prior to conducting the literature search. All identified articles were independently screened by two authors. Papers included in our review reported on clinical trials evaluating NNRTI-based treatment regimens in HIV-infected adults and administering at least one validated PRO instrument. Full-text articles of study abstracts which appeared to administer a PRO instrument were reviewed for the name and citation of the validated instrument. Reviews, editorials, animal studies, and those reporting results of children were excluded from our analysis.

### Data extraction

Data collected from each study included population characteristics, study design, study objective, treatments, and PRO instruments administered. We categorized each validated PRO instrument by type (e.g., HRQL, symptoms) to understand key domains of interest in NNRTI-based therapy. We also assessed the number of items, scoring, and dimensions/concepts measured by each instrument. For the most commonly used instruments, PRO-related data (e.g., baseline and follow-up scores, effect sizes, and significance values) were extracted from the studies, as available. The most commonly used PROs and study results were described.

## Results

A total of 189 articles were identified by the literature search and bibliography review. Most articles were excluded because they did not include a validated PRO instrument (n = 111). Articles were also excluded for one or more of the following reasons: review articles (n = 33), duplicate studies (n = 18), evaluated HIV therapies in children (n = 5), or did not evaluate NNRTI-based regimens (n = 13). Twenty-six unique clinical trials met all selection criteria and were included in the review.

Table 1 presents the characteristics of the 26 clinical trials. Almost all were randomized controlled trials (n = 20). The number of PRO instruments per study ranged from 1 to 7, with most studies including only one (54%) or two (23%) validated PRO instruments. In addition to validated PRO instruments, eight of the 26 trials (31%) used non-validated and study-specific instruments to measure such aspects as treatment preference, treatment satisfaction, perceived ease of regimen, and neuropsychiatric symptoms.

**Table 1:** Characteristics of included studies

**Study reference**	**Population characteristics**^**1**^	**Study design**	**Objective**	**Treatment groups**	**PRO instruments**^**2 **^**(validated, *not validated*)**	**Types of PRO instruments (validated only)**
Dabaghzadeh (2013) [7]	No prior EFV treatment experience (n = 51)	RCT (double-blind, placebo-controlled)	To assess the effect of cyproheptadine in prevention of neuropsychiatric adverse adverse drug reactions of ARV regimens containing EFV	1) ARV therapy (including EFV) + cyproheptadine	BDI-II	Psychiatric symptoms (3)
					HAM-A	
					HAM-D	Depression (2)
				2) ARV therapy (including EFV) + placebo	PANSS	Anxiety (1)
					PANSI	Sleep (1)
					PSQI	
					SCL-90 somatization subscale	
Bucciardini (2012) [8]	Treatment-experienced patients with treatment failure, resistance, or intolerance with HAART (2 NRTIs + NNRTI or PI) (n = 101)	Prospective, observational study (sub-set of ISS-NIA study)	To evaluate rates and determinants of virological failure in triple-class experienced patients receiving raltegravir-based regimens	1) All patients received RAL-based therapy; study compared patients with and without virologic failure on RAL	ISSQoL	HRQL: General (1)
Lake (2012) [9]	HIV-infected women with central adiposity and viral suppression on NNRTI- or PI-based HAART (n = 37)	RCT (open-label)	To evaluate effects of a switch from a PI or NNRTI to RAL on adipose tissue volume and metabolic changes	1) Immediate switch of PI or NNRTI to RAL (continuing prior NRTI backbone)	CES-D	Depression (1)
					Body Image Impact scale	Psychiatric symptoms (1)
				2) Delayed switch (at 24 weeks) of PI or NNRTI to RAL (continuing prior NRTI backbone)		
Mosam (2012) [10]	Treatment-naïve patients with HIV-associated Kaposi sarcoma (n = 112)	RCT (open-label)	To compare HRQL between 2 ARV regimens: ZDV/3TC/NFV versus ZDV/3TC/NVP	1) d4T/3TC/NVP	EORTC QLQ-30	HRQL: General (1)
				2) d4T/3TC/NVP + chemotherapy		
Cooper (2011) [11]	Treatment-experienced patients on stable ZDV/3TC/EFV regimen (n = 234)	RCT (open-label)	To assess the effect of switching ZDV/3TC/EFV to TDF/FTC/EFV on adherence, beliefs about ARV therapy and HQRL	1) Continue ZDV/3TC/EFV twice daily	BMQ-ART	HRQL: General (1)
					HAART Intrusiveness Scale	Medication beliefs: HIV-targeted (2)
				2) Switch to TDF/FTC/EFV once daily	SF-12 (v2)	
		
Nguyen (2011) [12]	Stable EFV-containing HAART regimen (n = 53)	RCT (double-blind, cross-over)	To investigate the effect of replacing EFV with RAL on patient preference, sleep quality, daytime sleepiness, anxiety, and lipid levels	1) Continue EFV-containing regimen, then switch EFV to RAL (continuing prior NRTI backbone)	ESS	Sleep (3)
					GSQS	
					SSS	
				2) RAL + prior NRTI backbone, then switch RAL to EFV (continue prior NRTI backbone)	*Treatment preference*	
					*Treatment satisfaction*	
Nguyen (2011) [13]	Stable EFV-containing HAART regimen (n = 58)	RCT (double-blind, cross-over)	To investigate the effect of replacing EFV with ETR on patient preference, sleep, anxiety, and lipid levels	1) EFV-based therapy	ESS	Sleep (3)
				2) ETR-based therapy	SSS	Anxiety/depression/stress (1)
					GSQS	
					DASS21	
					*Treatment preference*	
					*Treatment satisfaction*	
Campo (2010) [14]	PI-based HAART regimen without history of virological failure (n = 262)	RCT (open-label)	To evaluate the efficacy, safety and PROs of regimen switching to EFV-based HAART	1) Switch to EFV/3TC/ddI	FAHI	HRQL: HIV (1)
				2) Switch PI to EFV (continuing prior NRTIs)	IIRS	HRQL: General chronic disease (1)
					*Treatment preference*	
					*Treatment satisfaction*	
Cella (2010) [15]	Stable, but virologically failing ARV regimen (n = 1,203)	RCT (pooled analysis of DUET-1 and DUET-2)	To study the effects of etravirine versus placebo on the HRQL of HIV-infected patients	1) ETR 200 mg twice-daily^3^	FAHI	HRQL: HIV (1)
				2) Placebo^3^		
Cooper (2010)] [16]	HIV-infected, treatment-naïve patients (n = 87)	RCT (open-label)	To determine the impact of once-nightly versus twice-daily dosing and beliefs about HAART on adherence to EFV-based HAART in ARV-naïve patients	1) ddI/3TC/EFV once nightly	BMQ-ART	Medication beliefs: HIV-targeted (2)
				2) AZT/3TC twice daily + EFV nightly	HAART Intrusiveness Scale	
Cooper (2010) [17]; Regnault (2009) [18]	HIV-infected treatment-naïve patients (n = 895)	RCT (double-blind) [MERIT]	To evaluate the long-term efficacy, safety, adherence, and HRQL of once-daily EFV-based HAART	1) ZDV/3TC + MVC 300 mg twice daily	HIV-SI/SDM	HIV symptoms (1)
				2) ZDV/3TC + MVC 600 mg once daily		
				3) ZDV/3TC + EFV 600 mg once daily		
Hodder (2010) [19]; DeJesus (2009) [20]	PI- or NNRTI-based ARV regimen with virologic suppression (n = 300)	RCT (open-label)	To evaluate the therapeutic switch to a single-tablet formulation of EFV/FTC/TDF among virologically suppressed, HIV-infected adults	1) EFV/FTC/TDF	SF-36 (v2)	HRQL: General (1)
				2) Continue baseline ARVs (PI- or NNRTI-based)	HIV-SI/SDM	HIV symptoms (1)
					*Treatmet preference*	
					*Perceived ease of regimen*	
Potard (2010) [21]	Treatment experienced, NNRTI-naïve (n = 239)	Prospective, observational study	To assess changes in HRQL after switching to an NNRTI-containing regimen	1) EFV-based therapy	HADS	Anxiety/depression (1)
				2) NVP-based therapy	HIV-SI/SDM	
					WHOQOL-HIV	HIV symptoms (1)
					BREF	HRQL: General (2)
					SF-12 (v2)	
Clifford (2009) [22]	Treatment-naïve; study reports long-term follow-up of patients after unblinding of the AZT/3TC/ABC treatment arm (n = 303)	RCT (secondary analysis of A5095)	To evaluate the long-term impact of EFV-based regimens on neuropsychological performance	1) AZT/3TC/EFV	CES-D	Depression (1)
				2) AZT/3TC/ABC	PSQI	Sleep (1)
					STAI	Anxiety (1)
					*Neuropsychiatric symptoms*	
Gutierrez-Valencia (2009) [23]	Patients scheduled to receive an EFV-containing treatment plus 2 NRTIs (n = 114)	RCT (double-blind)	To determine if starting EFV in a stepwise dose schedule decreases EFV-related neuropsychiatric adverse events while maintaining efficacy	1) EFV-based therapy (stepwise dosing)	OSQ	Sleep (1)
				2) EFV-based therapy (full dose)	*Neuropsychiatric symptoms*	
Jayaweera (2009 ) [24]	Treatment-experienced patients (n = 65)	Prospective, single-arm trial (open-label) [DART I]	To evaluate the long-term efficacy, safety, adherence, and HRQL of once-daily EFV-based HAART	1) ddI/3TC/EFV once-daily	MOS-HIV	HRQL: HIV (1)
Jayaweera (2009) [24]	Treatment-experienced patients (n = 70)	Prospective, single-arm trial (open-label) [DART II]	To evaluate the long-term efficacy, safety, adherence, and HRQL of once-daily EFV-based HAART	1) d4T/3TC/EFV once-daily	MOS-HIV	HRQL: HIV (1)
Boyle (2008) [25]	Treatment-experienced patients on stable twice-daily or more frequent HAART (n = 320)	RCT (open-label)	To evaluate the effect of regimen simplification on maintenance of virologic suppression and treatment adherence	1) Continue baseline ARVs (BID + dosing)	FAHI	HRQL: HIV (1)
					IIRS	HRQL: General (1)
				2) Switch to once-daily d4T/3TC/EFV	*Treatment preference*	
					*Treatment satisfaction*	
DeJesus (2008) [26]	Stable regimen of fixed-dose AZT/3TC with EFV, experiencing AZT/3TC-related adverse effects or who might benefit from a simplified regimen (n = 402)	Prospective, single-arm trial	To evaluate the impact of switching from twice-daily AZT/3TC to once-daily TDF/FTC with EFV)	1) Switch from twice-daily AZT/3TC to once-daily TDF/FTC with EFV	SF-36 (v2)	HRQL: General (1)
					HIV-SI/SDM	HIV symptoms (1)
					*Treatment satisfaction*	
Bucciardini (2007) [27]	Treatment-naïve (n = 139)	RCT (secondary analysis of INITIO-QoL data)	To detect differences in patient’s HRQL among the 3 study treatment groups in the INITIO trial	1) ddI/d4T/EFV	MOS-HIV	HRQL: HIV (1)
				2) ddI/d4T/NFV		
				3) ddI/d4T/EFV/NFV		
Lafaurie (2008) [28]	NNRTI-naïve, receiving stable HAART consisting of at least 1 PI, 1 NRTI and AZT (n = 158)	RCT (open-label; secondary analysis of ALIZE data)	To assess if patients who have tolerated long-term AZT regimens will benefit from a switch to EFV/ddI/FTC	1) Maintenance of stable PI-containing regimen	MOS-HIV	HRQL: HIV (1)
				2) Switch to once-daily EFV/ddI/FTC		
Journot (2006) [29]	NNRTI-naïve, receiving unchanged HAART for ≥6 months consisting of at least 1 PI and 2 NRTIs (n = 355)	RCT (open-label; secondary analysis of ALIZE data)	To determine whether EFV use is associated with a higher incidence of depressive disorders compared to PI-containing regimens	1) Continue PI-based therapy	CES-D	Depression (1)
				2) Switch to EFV-based therapy		
Portsmouth (2005) [30]	Treatment-experienced patients with virologic suppression receiving d4T/3TC/EFV or ZDV/3TC/EFV (n = 43)	RCT (open-label)	To assess whether virologically controlled HIV-1-infected individuals switched from a twice-daily antiretroviral regimen to a once-daily regimen demonstrate improved adherence and quality of life while maintaining virological control	1) Continue twice-daily regimen of d4T(IR)/3TC/EFV or ZDV/3TC/EFV	MOS-HIV	HRQL: HIV (1)
				2) Switch to once-daily d4T(PRC)/3TC/EFV		
Casado (2004) [31]	Treatment-naïve; subset of patients with HRQL data in original COMBINE trial (n = 127)	RCT (secondary analysis of COMBINE)	To compare HRQL between 2 ARV regimens: ZDV/3TC/NFV versus ZDV/3TC/NVP	1) ZDV/3TC/NFV	MOS-HIV	HRQL: HIV (1)
				2) ZDV/3TC/NVP		
Negredo (2004) [32]	HAART experienced patients with long-lasting viral suppression (n = 169)	Prospective, observational study	To explore the long-term safety, and the virological and immunological efficacy of once-daily ddI/TDF/NVP in previously HAART-experienced subjects with long-lasting viral suppression	1) Continue twice-daily ARV therapy (PI- or NNRTI-based)	MOS-HIV	HRQL: HIV (1)
				2) Switch to once-daily ddI/TDF/NVP		
van Leth (2004) [33]	Treatment-naïve; subset of patients with HRQL data in original 2NN clinical trial (n = 917)	RCT (secondary analysis of 2NN data)	To investigate whether these differences in the safety profiles of EFV and NVP translates into differences in HRQL	1) d4T/3TC/EFV	MOS-HIV	HRQL: HIV (1)
				2) d4T/3TC/NVP		
				3) d4T/3TC/EFV/NVP		

*Abbreviations:* 3TC lamivudine, ABC abacavir, ACTG AIDS Clinical Trials Group, ARV antiretroviral, AZT zidovudine, BDI-II Beck Depression Inventory, second edition, BMQ-ART Beliefs about Medicines Questionnaire, adapted for antiretroviral therapy, CES-D Centre for Epidemiologic Studies-Depression scale, d4T stavudine, DASS21 Depression Anxiety and Stress Scale-short version, ddI didanosine, EFV efavirenz, EORTC QLQ-30 European Organization for Research and Treatment of Cancer Quality of Lfe Questionnaire, ESS Epworth Sleep Score, ETR etravirine, FAHI Functional Assessment of HIV Infection, FTC emtricitabine, GSQS Groningen Sleep Quality Score, HAART highly-active antiretroviral therapy, HADS Hospital Anxiety and Depression Scale, HAM-A Hamilton Anxiety Rating Scale, HAM-D Hamilton Depression Rating Scale, HIV-SI HIV Symptom Index, HRQL health-related quality of life, IIRS Illness Intrusiveness Rating Scale, ISSQoL Istituto Superiore di Sanità Quality of Life, MOS-HIV Medical Outcomes Study HIV health survey, NFV nelfinavir, NNRTI non-nucleoside reverse transcriptase inhibitor, NRTI nucleoside/nucleotide reverse transcriptase inhibitor, NVP nevirapine, PANSI Positive and Negative Syndrome Scale, PANSS Positive and Negative Suicide Ideation, PI protease inhibitor, PRO patient-reported outcome, PSQI Pittsburgh Sleep Quality Index, RCT randomized controlled trial, SCL-90 Symptom Checklist-90, SDM Symptom Distress Module, SF-12 MOS 12-item short-form health survey, SF-36 MOS 36-item short-form health survey, SSS Stanford Sleepiness Scale, STAI State-Trait Anxiety Inventory, TDF tenofovir, WHOQOL-HIV BREF World Health Organization Quality of Life-HIV, short version.

^1^All subjects are HIV-infected adults; ^2^Excludes patient-reported adherence-only instruments (e.g., ACTG Adherence Questionnaire); ^3^Both groups received darunavir/ritonavir (DRV/r) and an investigator-selected optimized background regimen of at least 2 ARVs consisting of NRTI(s).

The PRO instruments used corresponded to each study’s primary objective (e.g., HRQL studies used general or HIV-targeted HRQL instruments, a study to compare depressive symptoms in patients taking EFV- versus PI-based regimens used the CES-D, a depression-specific PRO instrument, etc.). Most studies utilizing a generic HRQL instrument (e.g., SF-36, SF-12) also included either an HIV-targeted HRQL or symptom instrument [11,14,19-21,25].

Overall, 27 validated PRO instruments were identified. Six of the instruments the Medical Outcomes Study HIV Health Survey (MOS-HIV), Functional Assessment of HIV Infection (FAHI), World Health Organization Quality of Life HIV BREF (WHOQOL-HIV BREF), HIV Symptom Index (HIV-SI)/AIDS Clinical Trials Group Symptom Distress Module (SDM), Beliefs about Medicines Questionnaire-ART version (BMQ-ART) and HAART Intrusiveness Scale were developed specifically to be administered in the HIV population. The remaining instruments were either generic HRQL instruments or general symptom-specific instruments.

Characteristics of the PRO instruments, including the number of items, concepts measured, and scoring method, are presented in Table 2. Based on review of the concepts measured by the PROs, key areas of interest measured by PROs in NNRTI clinical trials include general and HIV-targeted HRQL (typically comprised of physical, emotional, social, and functioning domains), HIV-related symptoms (including anxiety, depression, sleep, psychiatric symptoms, and stress), and medication-related beliefs.

**Table 2:** Characteristics of identified PRO measures

**Name**	**Instrument type**	**Items (N)**	**Domains/scales/concepts**	**Score type(s)**		
				**Dimension**	**Summary**^**1**^	**Total**
BDI-II	Psychiatric symptoms^2^	21	Severity of depression			X
BMQ-ART	Medication beliefs (HIV-targeted)	19	HAART necessity scale (beliefs about personal need for HAART for controlling HIV, maintaining their health, preventing illness), HAART concerns scale (potential adverse effects, dependence, embarrassment about treatment, etc.)	X		
Body Image Impact	Psychiatric symptoms	3	Belly size, belly image distress, belly profile	X		
CES-D	Psychiatric symptoms^2^	20	Frequency and severity of depression symptoms			X
DASS21	Psychiatric symptoms^2,3,4^	21	Depression, anxiety, stress	X		
EORTC QLQ-30	HRQL (general)	30	6 functioning scales (physical, role, cognitive, emotional, social, global QOL), 9 symptom scales/items (fatigue, pain, nausea and vomiting, dyspnea, sleep disturbance, appetite loss, constipation, diarrhea, financial impact)	X		
ESS	Sleep^5^	8	Rates chances of dozing during the daytime in 8 situations			X
FAHI	HRQL (HIV-targeted)	47	Physical well-being, functional and global well-being, emotional well-being/living with HIV, social well-being, cognitive functioning	X		X
GSQS	Sleep^6^	15	Questions about quality of previous night’s sleep			X
HAART Intrusiveness Scale	Medication beliefs (HIV-targeted)	12	Degree to which ART is perceived to interfere with aspects of daily life (e.g., social life, ability to work, relationships)			X
HADS	Psychiatric symptoms^2,3^	14	Anxiety, depression	X		
HAM-A	Psychiatric symptoms^3^	14	Severity of anxiety			X
HAM-D	Psychiatric symptoms^2^	17	Severity of depression			X
HIV-SI / SDM	HIV symptoms	20	HIV- or treatment-related symptoms (e.g., fatigue, dizziness, nausea, depression, anxiety)		X^7^	X
IIRS	HRQL (general)	13	Relationships and personal development, intimacy, instrumental	X		X
ISSQoL	HRQL (HIV-targeted)	62	QOL core (satisfaction with QOL, physical well-being, role well-being, social functioning, depression/anxiety, energy/vitality, health distress, cognitive functioning, sexual life), Additional important areas (social support, interaction with medical staff, treatment impact, body changes, life planning, motherhood/fatherhood)	X		
MOS-HIV	HRQL (HIV-targeted)	35	General health perceptions, physical functioning, role functioning, social functioning, pain, energy/fatigue, health distress, mental health, cognitive functioning, and quality of life	X	X^1^	X
OSQ	Sleep^6^	13	Subjective sleep quality, somnolence, insomnia, nightmares			X
PANSI	Psychiatric symptoms	14	Positive suicidal ideation, negative suicidal ideation	X		
PANSS	Psychiatric symptoms	30	Positive items (e.g., delusions, hallucinations), Negative items (e.g., blunted affect, emotional withdrawal), General Psychopathology (e.g., anxiety, depression, disorientation)	X		X
PSQI	Sleep^6^	19	Subjective sleep quality, sleep latency, sleep duration, habitual sleep efficiency, sleep disturbances, use of sleeping medication, daytime dysfunction			X
SCL-90 Somatization subscale	Psychiatric symptoms	12	Distress arising from perceptions of bodily dysfunction, such as cardiovascular, gastrointestinal, respiratory, and autonomic symptoms	X		
SF-12	HRQL (general)	12	Physical functioning, role physical, bodily pain, general health, vitality, social functioning, role emotional, mental health	X	X^1^	
SF-36	HRQL (general)	36	Physical functioning, role-physical, bodily pain, general health, vitality, social functioning, role-emotional, mental health, reported health transition	X	X^1^	
SSS	Sleep^5^	1	Subjects select 1 statement to best describe typical sleepiness at work during the prior week			X
STAI	Psychiatric symptoms^3^	40	State anxiety, trait anxiety			X
WHOQOL-HIV BREF	HRQL (HIV-targeted)	31	Physical, psychological, level of independence, social relationships, environment, spirituality	X		

*Abbreviations:* BDI-II Beck Depression Inventory, second edition, BMQ-ART Beliefs about Medicines Questionnaire, adapted for antiretroviral therapy, CES-D Centers for Epidemiological Studies-Depression, DASS21 Depression Anxiety and Stress Scale, short version, EORTC QLQ-30 European Organization for Research and Treatment of Cancer Quality of Life Questionnaire, ESS Epworth Sleep Score, FAHI Functional Assessment of HIV Infection, GSQS Groningen Sleep Quality Score, HAART Highly Active Antiretroviral Therapy, HADS Hospital Anxiety and Depression Scale, HAM-A Hamilton Anxiety Rating Scale, HAM-D Hamilton Depression Rating Scale, HIV-SI HIV Symptom Index, HRQL health-related quality of life, IIRS Illness Intrusiveness Rating Scale, ISSQoL Istituto Superiore di Sanità Quality of Life, MOS-HIV Medical Outcomes Study-HIV, OSQ Oviedo Sleep Questionnaire, PANSI Positive and Negative Syndrome Scale, PANSS Positive and Negative Suicide Ideation, PSQI Pittsburgh Sleep Quality Index, SCL-90 Symptom Checklist-90, SDM Symptom Distress Module, SF-12 MOS 12-item short-form health survey, SF-36 MOS 36-item short-form health survey, SSS Stanford Sleepiness Scale, STAI State-Trait Anxiety Index, WHOQOL-HIV BREF World Health Organization Quality of Life-HIV, short version.

^1^Physical health and mental health summary scores; ^2^Depression; ^3^Anxiety; ^4^Stress; ^5^Daytime sleepiness; ^6^Sleep quality; ^7^Symptom count and symptom bother count.

Table 3 provides a summary of the validated PRO instruments, categorized by instrument type, utilized in the 26 studies. The MOS-HIV, administered in 8 clinical trials, was the most commonly used PRO instrument. Table 4 presents PRO results for all PRO instruments used in three or more studies: the MOS-HIV, FAHI, HIV-SI/SDM, and CES-D.

**Table 3:** PRO instruments identified in trials with NNRTIs

**Instrument type and name**	**Study count**
**Instruments measuring quality of life**	
**Generic**	
SF-36 (v2)	2
SF-12 (v2)	2
EORTC QLQ-30	1
Illness Intrusiveness Rating Scale (IIRS)	2
Istituto Superiore di Sanità Quality of Life (ISSQoL)	1
**HIV-targeted**	
Medical Outcomes Study HIV (MOS-HIV)	8^1^
Functional Assessment of HIV Infection (FAHI)	3
World Health Organization Quality of Life (WHOQOL)-HIV BREF	1
**Instruments measuring symptoms**	
**General HIV symptoms**	
HIV Symptom Index (HIV-SI) /AIDS Clinical Trials Group Symptom Distress Module (SDM)	3^2^
**Sleep**	
Pittsburgh Sleep Quality Index (PSQI)	2
Epworth Sleep Score (ESS)	2
Stanford Sleepiness Scale (SSS)	2
Groningen Sleep Quality Score (GSQS)	2
Oviedo Sleep Questionnaire (OSQ)	1
**Anxiety (only)**	
Hamilton Anxiety Rating Scale (HAM-A)	1
State-Trait Anxiety Inventory for Adults (STAI)	1
**Depression (only)**	
Centers for Epidemiological Studies-Depression (CES-D)	3
Beck Depression Inventory-2nd edition (BDI-II)	1
Hamilton Depression Rating Scale (HAM-D)	1
**Anxiety and depression (only)**	
Hospital Anxiety and Depression Scale (HADS)	1
**Anxiety, depression, and stress**	
Depression Anxiety and Stress Scale-short version (DASS21)	1
**Psychiatric symptoms**	
Positive and Negative Syndrome Scale (PANSS)	1
Positive and Negative Suicide Ideation (PANSI)	1
Symptom Checklist-90 (SCL-90) - Somatization Subscale	1
Body Image Impact scale	1
**Instruments measuring beliefs about medications**	
Beliefs about Medicines Questionnaire-ART version (BMQ-ART)	2
HAART Intrusiveness Scale	2

^1^2 additional studies used modified versions of the MOS-HIV; ^2^2 additional studies used the ACTG Adherence Questionnaire, which contains the HIV-SI/SDM.

**Table 4:** PRO results of commonly used instruments

**Study reference**	**Treatment/dosing regimen**	**Domain**	**Baseline score mean (SD)**	**Follow-up score[time, mean]**	**Effect size**	**Summary of PRO results**
**Instrument: *Centre for Epidemiologic Studies – Depression Scale (CES-D)***						
Lake (2012) [9]	Immediate switch of PI or NNRTI to RAL (continuing prior NRTI backbone)	Depression	NR	24 weeks, NR	N/A	● The CES-D was administered at 0, 4, 8, 12, 18, and 24 weeks, but patient-reported depression scores were not reported in this study.
	Delayed switch (at 24 weeks) of PI or NNRTI to RAL (continuing prior NRTI backbone)	Depression	NR	24 weeks, NR	N/A	
Clifford (2009) [22]	ZDV/3TC/EFV	Depression	12.2 (10.5)	184 weeks^1^, 10.1^a^	0.20	● In participants who continued EFV-based regimens, neuropsychological performance improvement from baseline was maintained over 3 years.
	ZDV/3TC/ABC	Depression	11.8 (10.5)	184 weeks^1^, 10.4	0.13	● There was statistically significant decrease in depression symptoms over the course of the study with the median score decline of 1.0 (*P* = 0.03).
	Various regimens (± EFV)	Depression	13.3 (11.1)	184 weeks^1^, 16.6	−0.30	● In the long-term EFV-treated group, the percent with CES-D scores >16 declined from 34.1% to 22.3% over the duration of the study.
	ZDV/3TC/ABC initially, then EFV added (± ABC)	Depression	13.8 (12.5)	184 weeks^1^, 8.6	0.42	
Journot (2006) [29]	PI-based therapy	Depression^2^	23%	48 weeks, 25%	N/A	● Proportion of patients with depression was approximately 24% at BL and remained stable during the 48 week follow-up with no difference between treatment arms, *P* = 0.65).
	EFV-based therapy	Depression^2^	25%	48 weeks, 24%	N/A	
				36 months, 24%	N/A	
						● Patients with a history of depression experienced depressive symptoms more frequently than those without such history (53% and 22% at week 48, respectively; *P* = 0.03).
**Instrument: *Functional Assessment of HIV Infection (FAHI)***						
Campo (2010) [14]	Switch to EFV/3TC/ddI	Total Score	130	48 weeks, 134^a^	N/A	● In the overall patient population, FAHI total score increased significantly from BL to week 48 (*P* < 0.001) and at every other time point; changes in total score were associated with improvements in the physical and emotional well-being domains (P < 0.001 for both).
	Switch PI to EFV (continuing prior NRTIs)	Total Score	132	48 weeks, 138^a^	N/A	● No significant between-group differences observed.
Cella (2010) [15]	ETR 200 mg twice-daily^3^	Total Score	121.7 (23.7)^5^	24 weeks, 127.3^a,b^	0.21	● The change in physical well-being, emotional well-being/living with HIV and total scores from BL to Week 24 were statistically different from zero for both groups, with statistically significant greater improvements observed in the ETR group.
	Placebo^3^	Total Score	120.9 (26.7)^5^	24 weeks, 124.0^a,b^	0.11	
Boyle (2008) [25]	Continue BL ARVs (BID+ dosing)	Total Score	130.4	48 weeks, NR	N/A	● A small improvement (5% or less) for the emotional well-being and a small reduction (9% or less) for functional and global well-being were observed at some time points in both arms; however, these were not considered clinically relevant, as the effect sizes were small.
	Switch to once-daily d4T/3TC/EFV	Total Score	131.4	48 weeks, NR	N/A	● No significant differences observed between arms.
**Instrument: *HIV Symptom Index / Symptom Distress Module (HIV-SI / SDM)***						
Hodder (2010) [19]	EFV/FTC/TDF	Dizziness	28%	4 weeks, 39%^a,b^	N/A	● Simplification from PI-based or NNRTI-based regimens to EFV/FTC/TDF was associated with transient worsening or emergence of dizziness and sustained improvements in several other HIV-related symptoms: diarrhea or loose bowel movements; bloating, pain or gas in the stomach, changes in body appearance, and problems having sex.
				48 weeks, 28%	N/A	
	Remain on BL antiretroviral regimen	Dizziness	27%	4 weeks, 25%^b^	N/A	
				48 weeks, 28%	N/A	
Potard (2010) [21]	EFV- or NVP-based therapy	Symptom Count	11.9 (9.1)	12 months, 9.0^a^	0.32	● Overall, there was a small improvement in HIV symptoms at 1 year (effect size 0.32).
		Symptom Bother Count	7.7 (5.9)	12 months, 6.0^a^	0.29	● An initial difference between groups in mean change in other symptoms, bothersome symptoms, and other bothersome symptoms observed at 1 month was not maintained at months 6 and 12.
Regnault (2009) [18]	ZDV/3TC + MVC 300 mg twice dailyZDV/3TC + MVC 600 mg once dailyZDV/3TC + EFV 600 mg once daily	Symptom Count	Mean score ranged from ~5 (European Romance group) to ~10 (Bantu group)	96 weeks, NR	N/A	● This study assessed the cross-cultural validity of the HIV-SI using pre-ARV treatment cross-sectional data of the MERIT trial.
						● Statistically significant differential item functioning between cultural groups was observed for 4 items: fatigue, fevers, anxiety, and headache.
		Symptom Bother Count	Mean score ranged from 10.08 (European Romance group) to 24.00 (Bantu group)	96 weeks, NR	N/A	● The authors concluded that the absence of meaningful explanations for statistically significant differences between cultural groups supports the cross-cultural validity of the HIV-SI versions used in the MERIT trial.
DeJesus (2008) [26]	Switch from twice-daily AZT/3TC to once-daily TDF/FTC with EFV	Symptom Count	NR	24 weeks, NR	N/A	● Significant differences were observed in the percentage of patients reporting the absence of the symptom at Week 24 compared to BL for 17 of the 20 items assessed.
		Symptom Bother Count	NR	24 weeks, NR	N/A	● Compared to BL, significantly more patients reported the absence of fatigue, absence of nausea and vomiting, absence of diarrhea, and absence of headache.
**Instrument: *Medical Outcomes Study HIV health survey (MOS-HIV)***						
Jayaweera (2009) [24]	ddI/3TC/EFV once-daily	Total Score	874	96 weeks, 924	N/A	● The overall MOS-HIV QoL score, which is the sum of all individual MOS-HIV scores (range: 0 to 1,100), significantly improved from BL (874) to Week 96 (924; *P* < 0.05).
Jayaweera (2009) [24]	d4T/3TC/EFV once-daily	Total Score	832	12 weeks, 880	N/A	● The overall MOS-HIV QoL score significantly improved from BL (832) to Week 12 (880; *P* < 0.05).
Lafaurie (2008) [28]	PI-containing regimen	PHS	56.5 (50.0-61.8)^3^	48 weeks, -1.04^4^	0.24	● The mean change from BL to week 48 in the PCS and MCS were −1.04 and +0.0 in the maintenance arm and −1.76 and +1.01 in the switch arm, respectively (*P* = 0.57 and 0.42).
		MHS	40.2 (33.8-45.3)^3^	48 weeks, 0.00^4^	0.00	
	EFV/ddI/FTC	PHS	57.4 (51.5-60.4)^3^	48 weeks, -1.76^4^	0.53	● Specific items such as physical functioning, social functioning, and emotional functioning remained unchanged in both treatment groups during follow-up.
		MHS	38.3 (33.4-43.6)^3^	48 weeks, 1.01^4^	−0.27	
Bucciardini (2007) [27]	ddI/d4T/EFV	PHS	50 (11)	3 years, 54.9	−0.45	● Similar results reported for follow-up at years 1 and 2 (data not shown).
		MHS	49 (10)	3 years, 49.5	−0.05	
	ddI/d4T/NFV	PHS	46 (13)	3 years, 50.9	−0.38	● During follow-up, an increase of PHS score was observed in all treatment arms (NS).
		MHS	48 (10)	3 years, 53.5	−0.55	
	ddI/d4T/EFV/NFV	PHS	48 (12)	3 years, 50.0	−0.17	● The MHS score of both NNRTI- and PI-based 3-drug regimens showed a trend toward improvement but remained substantially unchanged with the four-drug combination.
		MHS	50 (9)	3 years, 53.4	−0.38	
Portsmouth (2005) [30]	Continue twice-daily regimen of d4T(IR)/3TC/EFV or ZDV/3TC/EFV	Total Score	NR	24 weeks, NR	N/A	● There were no significant differences in quality of life between the IR and PRC arms based on overall (sum of 11 domains) change from BL to week 24.
		Cognitive Function	NR	24 weeks, NR^a^	N/A	● Both arms showed significant improvement in the cognitive function domain (P < 0.001) during the course of the study, based on BL cognitive scores at weeks 12 and 24.
	Switch to once-daily d4T(PRC)/3TC/EFV	Total Score	NR	24 weeks, NR	N/A	● Differences between groups were not observed.
		Cognitive Function	NR	24 weeks, NR^a^	N/A	
Casado (2004) [31]	ZDV/3TC/NFV	PHS	54.16 (8.97)	12 months, 52.79	−0.15	● In the ZDV/3TC/NVP arm, there were statistically significant changes in the PHS score (P < 0.01) and a trend toward statistically significant change in the MHS score (*P* = 0.07).
		MHS	45.72 (11.10)	12 months, 49.20	0.31	
	ZDV/3TC/NVP	PHS	50.95 (11.37)	12 months, 56.73^a^	0.51	
		MHS	43.78 (9.92)	12 months, 48.22	0.45	● There were no statistically significant changes over time in the ZDV/3TC/NFV arm in both summary scores.
Negredo (2004) [32]	Continue twice-daily ARV therapy (PI- or NNRTI-based)	General Health	NR	NR	N/A	● Although quality of life tended to increase in both groups, no significant differences were found during the study in general health and health transition scales.
		Health Transition	NR	NR	N/A	
	Switch to once-daily ddI/TDF/NVP	General Health	NR	NR	N/A	
		Health Transition	NR	NR	N/A	
van Leth (2004) [33]	d4T/3TC/EFV	PHS	50.5	48 weeks, 53.9	N/A	● PHS and MHS BL values were comparable in all 3 treatment groups (*P* = 0.883 and *P* = 0.937, respectively).
		MHS	46.9	48 weeks, 53.9	N/A	
	d4T/3TC/NVP	PHS	51.0	48 weeks, 54.9	N/A	● No significant differences between the 3 treatment groups in increases in dimension scores.
		MHS	46.7	48 weeks, 52.8	N/A	● After adjusting for all significantly associated variables, the increase of PHS was 4.6 for NVP, 4.8 for EFV and 3.8 for NVP + EFV (*P* = 0.790); the adjusted increase of MHS was 6.1, 7.3 and 3.8, respectively (*P* = 0.093).
	d4T/3TC/EFV/NVP	PHS	50.9	48 weeks, 53.8	N/A	
		MHS	47.1	48 weeks, 51.0	N/A	

*Abbreviations: 3*TC lamivudine, ABC abacavir, ARV antiretroviral, AZT zidovudine, BL baseline, CF cognitive functioning, d4T stavudine, ddI didanosine, DRV/r darunavir/ritonavir, EFV efavirenz, ENF enfuvirtide, EWB emotional well-being, ETR etravirine, FGWB functional global well-being, FTC emtricitabine, HAART highly-active antiretroviral therapy, IQR interquartile range, IR immediate release, MCS mental component score, MHS mental health summary, N/A not available, NFV nelfinavir, NNRTI non-nucleoside reverse transcriptase inhibitor, NRTI nucleoside/nucleotide reverse transcriptase inhibitor, NS not significant, NVP nevirapine, PCS physical component score, PHS physical health summary, PI protease inhibitor, PRC prolonged-release capsule, PRO patient-reported outcome, PWB physical well-being, SD standard deviation, SWB social well-being, TDF tenofovir, ZDV zidovudine.

^1^184 weeks, or study discontinuation; ^2^Score ≥17 for men and ≥23 for women; ^3^Median (IQR); ^4^Mean change; ^5^Standard deviation estimated from IQR.

^a^Significant change over time (*P <* 0.05); ^b^Significant change between groups (*P* < 0.05).

## Discussion

Evaluation of PROs during clinical practice, as well as in clinical research, enhances understanding of disease impact and effect of treatment on that disease impact. Thus, PRO assessment should be recognized by patients and their physicians, as well as by payers and health technology assessment authorities, as improving the knowledge base on which to base health care decision making, and ultimately to improve patient health. This study found that the key areas of PRO interest in clinical trials of NNRTI-based therapy are HRQL (general or HIV-targeted, and typically comprised of physical, emotional, social and functioning domains), HIV symptoms, sleep, and psychiatric symptoms, including anxiety, depression, stress, and medication beliefs. A variety of instruments were used to measure these dimensions. The only instruments used in three or more clinical trials within the past ten years were the MOS-HIV, FAHI, and CES-D.

Overall, although we were able to identify important concepts measured in NNRTI studies based on the convergence of PRO instrument types (e.g., HRQL, HIV symptoms, anxiety, depression), there was a noticeable lack of consensus among studies on specific instruments utilized to measure each concept. For example, of five generic HRQL instruments identified, none were used in more than two studies.

To our knowledge, this is the first study to systematically identify and categorize PRO instruments used specifically in NNRTI clinical trials. Clinical trials commonly use more than one PRO instrument. Although each PRO instrument may be able to contribute valuable information, it is important to carefully weigh the advantages and disadvantages of each instrument, especially related to its sensitivity and specificity to capture the patient factors of greatest importance. This is important both maximize the chances of detecting important differences between treatments, as well as to limit patient response burden.

A multidimensional generic HRQL instrument, such as the SF-36 or EQ-5D, is useful because it comprehensively measures HRQL and has norm-based scoring which can be used to compare the study population with others. Furthermore, it can be used in population-wide decision making by providing data on quality of life weights or utilities for inclusion in cost-effectiveness and cost-utility analyses. For example, this can be done directly (e.g., using the EQ-5D) or indirectly (e.g., by deriving SF-6D utility weights from the SF-36). However, a disadvantage of using generic measures is that they may be less sensitive or responsive to small but important changes that occur due to changes in disease status, adverse events, or to treatment effect, and which may occur over the typical timeframe of a randomized control trial.

HIV-targeted HRQL instruments, such as the MOS-HIV, FAHI, and WHOQOL-HIV BREF, were each developed by revising, at least in part, generic HRQL instruments (the SF-20, Functional Assessment of Cancer Therapy-General [FACT-G], and WHOQOL-BREF, respectively) with input from HIV-infected patients and HIV-treatment providers to ensure more complete coverage of concepts specific to HIV infection. Each instrument demonstrates excellent psychometric properties in the HIV population. In contrast to the generic HRQL instruments, a disadvantage of HIV-targeted instruments is that they do not provide a means for estimating utilities, which can be useful in clinical-economic modeling considered by health technology assessment authorities and others focused on population health.

For HIV-related symptoms, the HIV-SI/SDM is considered to be the gold standard in clinical research. However, a generic symptom-specific instrument may be more appropriate when the primary or secondary study objective is to measure a specific symptom; such symptom-specific instruments generally measure the symptom and different attributes and impacts with multiple items, thus providing greater insights into the extent and effect of the measure.

More than half of the articles initially identified were excluded from our review because the abstract did not report use of a PRO instrument. However, this likely underestimates of the frequency of administration of PRO instruments in clinical trials for two reasons: 1) we used an extensive list of search terms in order to capture as many validated PRO instruments as possible, and consequently identified non-relevant articles, and 2) PROs are generally secondary endpoints in clinical trials; as such, they may not be mentioned in the study abstract and commonly are reported in separate publications. Since we did not review the full text of excluded articles, we do not know if the excluded studies were unique clinical trials or secondary publications of identified trials.

There are several limitations to our study that should be noted. First, our review excluded questionnaires measuring adherence because we were only interested in patient-reported measures of treatment effects. However, it should be noted that the HIV-SI/SDM is a component of the ACTG Adherence Questionnaire, a validated instrument developed by the AIDS Clinical Trial Group. Although our review excluded studies which mentioned only adherence and no additional patient-reported measures in the study abstract, based on abstract review we identified two studies which used the ACTG Adherence Questionnaire [34,35]. It is possible that there are additional studies which used the ACTG Adherence Questionnaire as the adherence measure, and therefore also measured HIV symptoms with the HIV-SI/SDM, which were not included in our literature review. Secondly, our review only evaluated studies using validated PRO instruments. However, some studies use study-specific instruments which are based on one or more validated instruments. For example, studies by Santos et al. [36] and Martinez-Picado et al. [37] used modified versions of the MOS-HIV and thus were not fully evaluated in our review. Finally, our review focused on PRO instruments included in prospective clinical trials of NNRTIs. It should be noted that there are clinical research networks, such as the Centers for AIDS Research Network of Integrated Clinical Systems (CNICS) which allow for retrospective review of PROs measured during routine medical visits [38]. PRO instruments used at these clinical sites include the Patient Health Questionnaire (PHQ) for depression and anxiety, HIV-SI for symptom burden, and EQ-5D for HRQL, among others. For example, a study by Kozak et al. [39] used reports from the Patient Health Questionnaire depression scale (PHQ-9) and the Alcohol, Smoking and Substance Involvement Screening Test (ASSIST) to demonstrate that current substance abuse (odds ratio [OR], 2.78; 95% confidence interval [CI], 1.33–5.81) and current depression (OR, 1.93; 95% CI, 1.12–3.33) were associated with poor antiretroviral adherence in HIV patients. Additional research, including review of NNRTI studies published in non-English languages and retrospective analyses of PROs collected during usual medical care visits, should be conducted and could build on the findings presented here.

## Conclusions

Review of recently published NNRTI clinical trials suggests a lack of consensus on the optimal PRO instruments to include to measure key domains. Overall, a typical battery of instruments is comprised of a multidimensional measure of HRQL (either HIV-targeted or generic) coupled with one or more symptom measures. Further work is needed to clarify the advantages and disadvantages of using various instruments to measure the relevant constructs and to identify the most useful batteries of instruments. Furthermore, new instruments may need to be developed to meet future research needs.

## Competing interests

KAH and CLP are employees of UBC and GH is an employee of Evidera, both of which received funding for this research from Pfizer. SH and MT are employees of and have equity ownership in Pfizer. AK was an employee of Pfizer at the time the study was conducted. KNS and AWW received funding for this research from Pfizer.

## Authors’ contributions

KAH and GH participated in the study conception and design, acquisition of data, data analysis and interpretation, and manuscript writing. KNS, SH, MT, CLP, and AWW participated in the study conception and design, data interpretation, and manuscript writing. AK participated in the data interpretation and manuscript writing. All authors read and approved the final manuscript.

## Supplementary Material

Additional file 1 Table A Search terms for identifying clinical studies of NNRTIs with PRO instruments. Click here for file
