# Supplementary material for: Patient reported outcome instruments used in clinical trials of HIV-infected adults on NNRTI-based therapy: a 10-year review
Source: Health Qual Life Outcomes. 2013 Oct 3;11:164. doi: 10.1186/1477-7525-11-164 (PMC3852266; doi:10.1186/1477-7525-11-164)
Supplement: Additional file 1 — Table A Search terms for identifying clinical studies of NNRTIs with PRO instruments. [file 1477-7525-11-164-S1.doc]

**Additional file**

**Table A. Search terms for** identifying clinical studies of NNRTIs with PRO instruments

| **Category** | **Search string** |
| --- | --- |
| **HIV terms** | HIV[MeSH Terms] OR "HIV infections"[MeSH Terms] OR HIV[tiab] OR “human immunodeficiency virus”[tiab] |
| **NNRTI terms** | Efavirenz[tiab] OR Sustiva[tiab] OR EFV[tiab] OR Efavirenz[Substance Name] OR Nevirapine[tiab] OR Viramune[tiab] OR NVP[tiab] OR Nevirapine[MeSH Terms] OR Delavirdine[tiab] OR Rescriptor[tiab] OR DLV[tiab] OR Delavirdine[MeSH Terms] OR Etravirine[tiab] OR Intelence[tiab] OR ETV[tiab] OR Etravirine[Substance Name] OR Rilpavirine[tiab]OR TMC278[tiab] OR TMC-278[tiab] OR Rilpivirine[Substance Name] OR Atripla[tiab] OR "efavirenz, emtricitabine, tenofovir disoproxil fumarate drug combination "[Substance Name] OR Edurant[tiab] OR Complera[tiab] OR Lersivirine[tiab] OR non-nucleoside[tiab] OR NNRTI[tiab] |
| **Clinical trial terms** | trial[tiab] OR trials[tiab] OR Clinical Trial [Publication Type] OR Clinical Trial, Phase IV [Publication Type] OR Clinical Trial, Phase III [Publication Type] OR Clinical Trial, Phase II [Publication Type] OR Controlled Clinical Trial [Publication Type] OR Randomized Controlled Trial [Publication Type] |
| **PRO / instrument terms** | questionnaires[MeSH Terms] OR questionnaire[tiab] OR questionnaires[tiab] OR survey[tiab] OR surveys[tiab] OR instrument[tiab] OR instruments[tiab] OR scale[tiab] OR scales[tiab] OR diary[tiab] OR diaries[tiab] OR inventory[tiab] OR inventories[tiab] OR "Interviews as Topic"[MeSH Terms] OR interview[tiab] OR interviews[tiab] OR interviewed[tiab] OR Health Related Quality of Life[tiab] OR HRQL[tiab] OR HRQOL[tiab] OR "quality of life"[MeSH Terms] OR "quality of life"[tiab] OR QOL[tiab] OR well-being[tiab] OR “health assessment questionnaire”[tiab] OR “health assessment questionnaires”[tiab] OR Patient Satisfaction[MeSH Terms] OR "patient satisfaction"[tiab] OR "patient reported"[tiab] OR "self report"[tiab] OR "self reported"[tiab] OR “self reporting”[tiab] OR “patient preference”[tiab] OR “patient preferences”[tiab] OR “patient assessment”[tiab] OR “patient assessments”[tiab] OR “patient assessed”[tiab] OR “self evaluation”[tiab] OR “self evaluations”[tiab] OR “patient rating”[tiab] OR “patient ratings”[tiab] OR “patient rated”[tiab] OR self-completed[tiab] OR self-administered[tiab] OR “self assessment”[tiab] OR “self assessments”[tiab] OR “patient based outcome”[tiab] OR "Self-Evaluation Programs"[MeSH Terms] OR Euroqol[tiab] OR euro-qol[tiab] OR EQ-5D[tiab] OR MOS-HIV[tiab] OR "Medical Outcomes Study"[tiab] OR HADS[tiab] OR "hospital anxiety and depression scale"[tiab] OR HIV-SI[tiab] OR SDM[tiab] OR "Symptom Index" OR "symptom distress module"[tiab] OR UCLA-DQ[tiab] OR "dizziness questionnaire"[tiab] OR ESS[tiab] OR "Epworth Sleepiness"[tiab] OR ACTG[tiab] OR "adherence questionnaire" OR Hamilton[tiab] OR HAM-D[tiab] OR HAMD[tiab] OR Hamilton[tiab] OR HAM-A[tiab] OR HAMA[tiab] OR "Profile of Mood States"[tiab] OR POMS[tiab] OR "Assessment of body change distress"[tiab] OR ABCD[tiab] OR CPRCA[tiab] OR "health utilities index"[tiab] OR HUI[tiab] OR "Targeted quality of life"[tiab] OR HAT-QOL[tiab] OR "functional assessment of HIV"[tiab] OR FAHI[tiab] OR "beck depression"[tiab] OR BDI[tiab] OR rotterdam[tiab] OR "symptom checklist"[tiab] OR "cognitive capacity screening"[tiab] OR DMS-48[tiab] OR DMS[tiab] OR "behavior and symptom identification"[tiab] OR BASIS[tiab] OR Burke[tiab] OR BUPP[tiab] OR "dementia motor scale"[tiab] OR HDMS[tiab] OR "sense of coherence"[tiab] OR SOC[tiab] OR "social factors of antiretroviral therapy"[tiab] OR soc-fart[tiab] OR "brief pain inventory"[tiab] OR BPI[tiab] OR McGill[tiab] OR "pain questionnaire"[tiab] OR "pain inventory"[tiab] OR "dizziness handicap"[tiab] OR DHI[tiab] OR "functional status II"[tiab] OR FSII[tiab] OR "treatment satisfaction"[tiab] OR HIVTSQ[tiab] OR HIV-TSQ[tiab] OR "customized adherence"[tiab] OR "adherence determinants"[tiab] OR Leeds[tiab] OR "sleep evaluation"[tiab] OR "symptom checklist"[tiab] |
